# Seeing the Experimenter Influences the Response to Pointing Cues in Long-Tailed Macaques

**DOI:** 10.1371/journal.pone.0091348

**Published:** 2014-03-19

**Authors:** Vanessa Schmitt, Christian Schloegl, Julia Fischer

**Affiliations:** 1 Social Cognition Cologne, University of Cologne, Cologne, Germany; 2 Cognitive Ethology Laboratory, German Primate Center, Göttingen, Germany; 3 Courant Research Centre “Evolution of Social Behaviour”, Georg-August-University Göttingen, Göttingen, Germany; Institut Pluridisciplinaire Hubert Curien, France

## Abstract

Methodological variations in experimental conditions can strongly influence animals' performances in cognitive tests. Specifically, the procedure of the so-called object-choice task has been controversially discussed; here, a human experimenter indicates the location of hidden food by pointing or gazing at one of two or more containers. Whereas dogs usually succeed, results for nonhuman primates are ambiguous. In the standard version of the task the majority of subjects do not respond appropriately to human pointing. However, modifying the task setup, such as placing the containers further apart, seems to improve subjects' performances, suggesting that cue salience may be an important variable. Here we investigated whether the visibility of the experimenter inhibits long-tailed macaques' (*Macaca fascicularis*) usage of the pointing cue. In our baseline condition, with the experimenter fully visible, the monkeys chose the correct container in 61% of the trials. The performance increased significantly, however, when the experimenter was hidden behind a curtain and only the arm of the experimenter, a doll's arm, or a stick was visible. Furthermore, the monkeys performed significantly better when the tip of the pointing finger or device was close to the target compared to the more distant condition. Intriguingly, after these experiments the monkeys' performance was also significantly improved in the baseline condition (70%). Apparently, the monkeys were first distracted by the presence of the experimenter, but then learned to use the cue. These findings highlight the importance of the test conditions, and question some of the assumptions about species-specific differences in the object-choice task.

## Introduction

Methodological variations can strongly influence animals' performances in cognitive experiments. Monkeys, for example, can discriminate quantities much better when the reward and the choice stimuli are separate entities than when they have to choose between different food amounts only [1]. The field of comparative cognition heavily relies on testing the cognitive competences of different animal species [2], and it is essential to assure putative differences in performance are indeed due to species differences and not to slightly different methods.

One frequently used experiment in comparative cognition is the so-called object-choice task in which subjects are tested for the comprehension of communicative cues. In this experiment a human experimenter usually points at one of two (or more) cups or containers to indicate the location of a hidden reward (see [3], [4] for reviews on pointing). Gestures such as pointing with the index finger to a specific object, person or event are ubiquitously understood communicative actions in humans [5]. Whether nonhuman animals are also able to use these communicative cues and how this ability may have evolved is therefore a central question in socio-cognitive research [6]–[9]. Whereas dogs usually succeed (e.g. [8], [10]), and domesticated or highly trained subjects seem to perform better (e.g. domestic goats [11], horses [12], dolphins [13], fur seals [14], wolves [15]) the picture is more complicated in nonhuman primates. In some studies they exhibited considerable difficulties to use human communicative cues and performed inferior to dogs (e.g. [16]), but other studies reported that nonhuman primates did use human-pointing cues reliably in the object-choice task [6], [17].

The results from nonhuman primates raised the question of the suitability of the object-choice task to explore and compare animals' socio-cognitive abilities. In its standard version used with nonhuman primates, two cups are placed in-between the subject and the experimenter in relatively close distance to each other. In this setup most nonhuman primates fail to choose the cup pointed at (with the exception of highly enculturated apes [18]–[23]). Mulcahy and colleagues [6] developed a slightly different design and found that placing the cups further apart so that the experimenter was standing in between the cups and pointed at a cup located either on the left or right side of his body improved the performance substantially. In their so-called distraction hypothesis [9] the authors proposed that seeing both containers in close proximity and between themselves and the experimenter draws the subjects' attention towards the containers and away from the cue. In consequence, they may choose a container without even attending to the cue. They argued that a larger distance between the different containers is crucial for a successful performance, as it increases the salience of the communicative cues.

In addition to this distance effect, other factors may also influence primates' usage of human communicative cues in an object-choice paradigm. Rhesus macaques (*Macaca mulatta*) tested in the field rather than under standard lab conditions used human pointing cues to locate a food reward [24], [25]; likewise, a reduction in distance between the finger tip and the pointed-at container usually improves performances [e.g. [26], see [4] for a review). In principle the standard task is a cooperative task (the experimenter helps the subject by providing the cue), but chimpanzees performed significantly better in a competitive version, in which the experimenter and the chimpanzee competed by both reaching toward the reward [27]. This suggests that the relationship between the subject and the experimenter can influence the task performance. In fact, nonhuman primates may regard humans rather as competitors than as cooperation partners and an experimenter facing a subject may appear threatening for many species [27], [28]. Supporting this idea, studies with lemurs showed that they avoid a human facing them in object-choice tasks [29].

Furthermore, a closer look at the experimental setups suggests that in many studies subjects may solve the task by following simple associative rules such as “choose the container closest to the experimenter's hand”. Nevertheless, successful performance is often considered to demonstrate extraordinary socio-cognitive abilities, such as an understanding of others' minds (e.g. [10], [30]). But is this really necessary to solve the task? Recently, Elgier and colleagues [31] demonstrated that dogs' usage of human pointing cues shows many features of more simple associative learning. For instance, their behaviour was characterized by stimulus generalisation from learned to new cues, as well as typical extinction processes when varying the reward scheme. Furthermore, in their studies dogs did not show a general preference for social cues compared to physical ones, such as the colour of the cups ([31], see [32] for a review). As the underlying mechanisms of interspecific communication are still unclear, it remains controversial whether dogs or other animals understand the communicative intention of pointing or whether their performance is due to rapid learning [33], [34].

In this study we aimed to achieve a better understanding of the procedural constraints and cognitive mechanisms influencing the usage of human pointing cues in long-tailed macaques (*Macaca fascicularis*), a monkey species performing similar to apes in a range of physico- and socio-cognitive experiments [35]. By manipulating the presence of the experimenter, we set out to test the following non-mutually exclusive hypotheses regarding the use of the pointing cue. First, the monkeys may be distracted by the experimenters' body and face in the normal pointing paradigm. Thus, we ‘removed’ the body of the experimenter by placing her behind a curtain with only the pointing arm being visible to the subjects. If the presence of the body/face is reducing the ability to decide correctly, the monkeys should perform better with the experimenter standing behind the curtain. Second, the monkeys may still link the arm to the presence of the experimenter. We therefore also used a doll's arm and a stick to deliver the pointing cues to examine the influence of the quality of the cue-providing element; this should allow us to elucidate if a “relatively social” (the arm of the experimenter), an “abstract social” (the doll's arm) and an “abstract arbitrary” element (the stick) would influence the monkeys' performance differently. If the degree of abstractness enhances performance, we would predict that the monkeys' performance is best in the ‘stick’ condition, and intermediate in the ‘doll’ condition. Third, a considerable effect of local enhancement (distance between the cue and the container) regardless of condition, would further suggest that the animals do not take the communicative intent into account [26] but rather respond to associative rules. Last, the absence of the experimenter may facilitate general learning to understand the pointing cue. In this case, we would predict that the subjects' would be better able to use the pointing cue with the experimenter present after they had been tested in the modified tasks with the experimenter being removed.

## Materials and Methods

### Ethics Statement

All testing was non-invasive and the subjects participated voluntarily in the experiments. They were not food deprived for testing and water was always available ad libitum. The monkeys were fed regular monkey chow, fruits and vegetables twice a day. Their enclosure was equipped with wooden platforms, fire hoses, and several enrichment objects, which were changed on a regular basis. All experiments were performed under the control of experienced veterinarians to ensure that the studies were in accordance with the NRC Guide for the Care and Use of Laboratory Animals and the European Directive 2010/63/EU on the protection of animals used for scientific purposes. Furthermore, in accordance with the German Animal Welfare Act and corresponding section for animals used for scientific purposes, the study approval was checked by the responsible Animal Welfare Officer of the German Primate Center (Permit Number 33.9–42502).

### Subjects

We tested 10 long-tailed macaques - 4 males and 6 females aged 2 to 8 years - at the German Primate Center in Göttingen (one subject was only tested in the proximal baseline and test conditions because of health problems) living in a social group of 28 animals. The monkeys had access to indoor (25 sqm) and outdoor enclosures (141 sqm). They were individually tested in a small cage (58×42×57 cm, l×w×h) connected to a testing compartment (approx. 1 sqm, 2.3 m high) inside their familiar indoor area. The subjects were free to leave the cage and enter the testing compartment at any time during testing. Tests were conducted once or twice per day and participation was voluntary, that is, dependent on the monkeys' willingness to enter the test compartment. The subjects had already participated in a battery of tasks assessing their socio- and physico-cognitive capacities [1], [35]. Here they had also been tested in a pointing task; however, only few trials had been conducted and they had not performed above chance.

### Testing Apparatus

**Figure 1:**
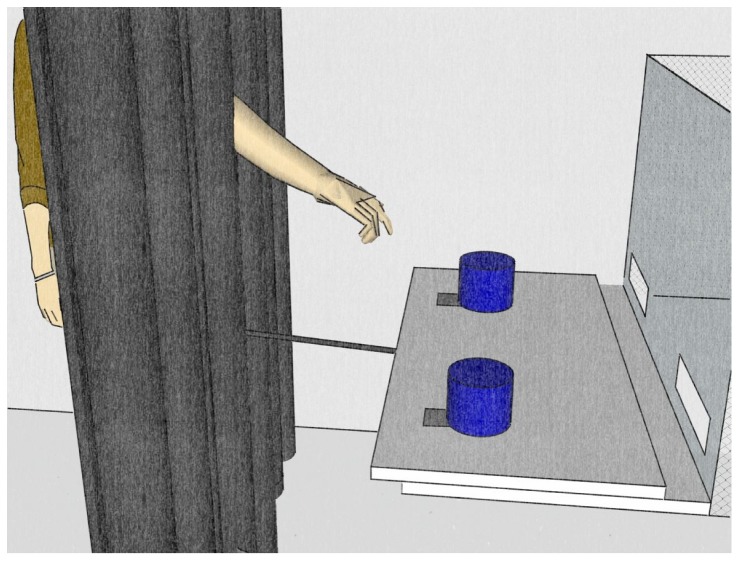
Simplified drawing of the test situation. The experimenter stood behind a curtain and only the arm, a doll's arm or a stick pointing at the baited cup was visible to the subject sitting in the test cage.

Two blue opaque cups (Ø 5 cm×5 cm height) were used to hide the reward (raisins or peanuts). The cups were fixed on a sliding board (distance 30 cm) with hinges so that the monkeys could lift them from the front revealing whether a food reward was hidden underneath or not. The sliding board was attached to a fixed table (length 55 cm, width 30 cm) so that the sliding board could be moved horizontally. The table was attached to a plastic panel, which had two oval openings at the outer sites (5.5 cm×2 cm, distance 30 cm) to allow the subjects to put one hand through and reach for a cup (see Figure 1).

To hide the experimenter we put up a large black curtain (2 m×1.50 m) in front of the testing apparatus. We cut a small hole into the middle of the curtain to allow inserting an arm or stick to point at a cup. Furthermore, we attached a baton to the end of the sliding board, which passed through the curtain and allowed to move the table back and forth from behind the curtain.

To point at the cups we either used the arm of the experimenter, a grey coloured stick (Ø 4 cm×80 cm length) or a left and right arm of a regular fashion mannequin (Ø 30 cm×80 cm length), referred to as doll in the remaining text. From behind the curtain it was possible to lower down an occluder of black cardboard (70 cm×100 cm), hanging from the ceiling, to prevent the subjects from seeing the experimenter during the baiting procedure. The experimenter coded live which cup the monkey chose first. The choice was always unambiguous as they only reached for one cup at a time. In addition, all sessions were videotaped (Sony DCR-HC90E) and a second observer coded 20 percent (N = 342 trials) of all videos resulting in a complete concordance with the initial coding.

### Experimental Procedure

Before the experiment started the tested subject was separated from the group in its familiar testing cage and received some food rewards to control for its general motivation to participate in the experiment. The experimenter then lowered down the occluder and baited one of two opaque cups out of view of the subject. In the test conditions the experimenter then stepped back behind a large curtain for the remaining session, remotely lifted the occluder from behind the curtain and put her arm, a stick, or the arm of the doll through a hole in the middle of the curtain to point at the baited cup. She always used the arm contralateral to the baited cup, i.e. the left arm to point at the right cup and vice versa (also called *cross pointing*, [4]). After approximately three seconds, she pushed the sliding table, while still pointing, to the monkey, which then could choose one of the cups. After the subject had made its choice and took the reward in case of a correct answer, the experimenter lowered the occluder again and re-baited the cups, leading to an inter-trial interval of approximately 30 seconds. The position of the reward was balanced with the restriction that it could not appear on the same side for more than two consecutive trials. Half of the animals first passed all conditions with proximal cueing (5–10 cm between cup and finger) and then received distal cueing (30-40 cm), the others received the opposite order to control for order effects. All animals received one or two sessions consisting of 9 trials (see below) per day (5 days a week). One session lasted about 10 to 15 minutes. If an animal was not willing to participate in a session (e.g. not choosing a cup), it was released to the group and tested on another day.

### Conditions and Design

#### Control and Familiarisation

To familiarise the animals to the setup, to the procedure of lifting the cups by themselves to retrieve the food, and to control if the animals used cues like smell to find the reward, we administered a condition in which the experimenter did not cue the correct location. She baited one cup behind the occluder, lifted the occluder, and just waited until the subject made its choice. Each subject received two 9-trial sessions.

#### Baseline

In the baseline condition the experimenter did not step behind the curtain and only pointed with her arm at the baited cup. Thereby she gazed straight forward, not looking at the monkey or the cups. Each subject received two 9-trial sessions with proximal cueing and two sessions with distal cueing (18 trials each).

#### Test

After baiting, the experimenter stepped behind the curtain and lifted the occluder. She then either put her own arm (*human*), the arm of a doll (*doll*), or a stick (*stick*) through the curtain to point at the baited cup.

Each subject received six 9-trial sessions (54 trials in total) with proximal and six 9-trial sessions with distal cueing. Each session contained 3 trials of each test condition (i.e. human, stick, doll) resulting in 18 trials per condition for the distal and for the proximal variant each. The order of conditions was pseudo-randomised and balanced within and across individuals.

#### Re-Test

After the completion of all experimental conditions, all subjects were re-tested, using the same procedure as in the baseline condition, with the experimenter present. Each subject received two 9-trial sessions with proximal and distal cueing each.

### Statistics

Data were analysed using Statistica 9.1 (StatSoft, Inc.) and SigmaPlot 11.0 (Systat Software, Inc.). As the data were not normally distributed (normality tests using Shapiro-Wilks) and the sample size was rather small, we used non-parametric statistics to analyse the data. We used exact, two-tailed Wilcoxon tests to determine whether the performance of the monkeys was above chance level (i.e. 50% correct) in the different conditions. We conducted Friedman ANOVAs to test whether the monkeys' performance differed between the four different conditions (baseline, doll, human, stick), in the proximal and distal cueing conditions. We conducted Post hoc tests (Holm-Sidak method) in case of a significant result. As one monkey participated in the proximal conditions only, we excluded her from this analysis, thus comparing the results of 9 monkeys only. To test whether the monkeys' performance had increased in the re-test condition, we compared these results to the baseline condition using Wilcoxon's exact test.

## Results

**Figure 2:**
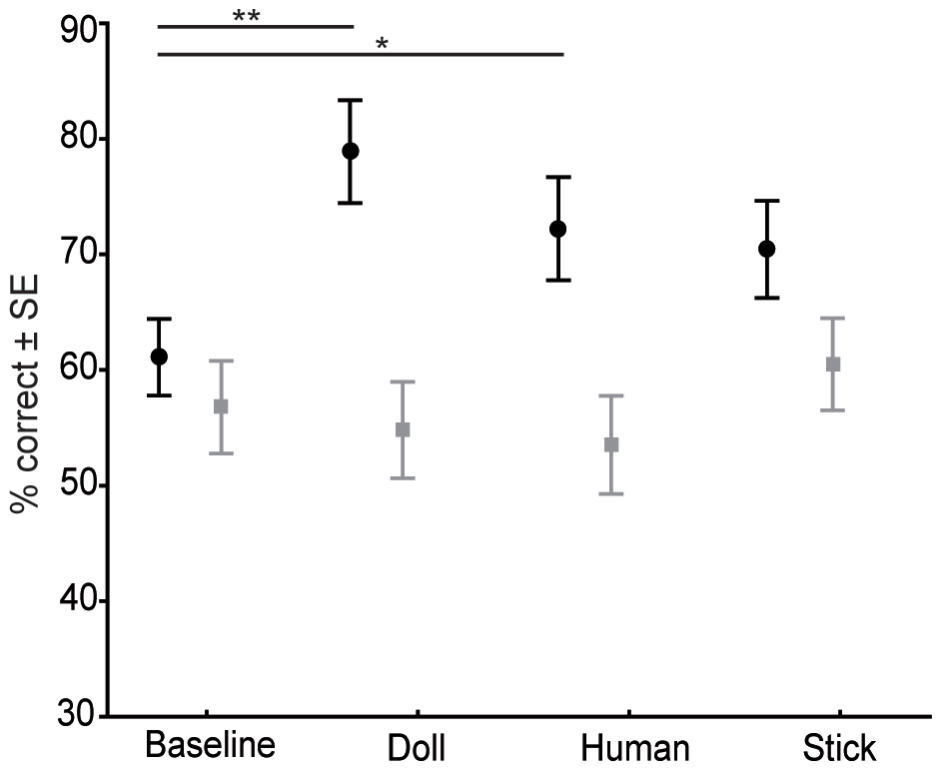
Performance of the long-tailed macaques in each condition. The figure shows the percentage of correct trials in each of the proximal (black circle) and distal (grey square) pointing conditions (means and standard error of means). The performance in the proximal Doll and Human condition was significantly better than in the proximal Baseline condition (Wilcoxon's exact test, baseline vs. doll **p<0.001; baseline vs. human *p = 0.033).

All subjects performed at chance level in the *control* condition (N = 10, Mean: 51.1%±0.016 SE correct, W = 9, p = 0.547) and thus did not use any cues like smell or sight to find the reward. In the *baseline* condition, the monkeys performed at chance level with distal cueing (N = 9, W = 12, p = 0.125) and only slightly better than chance with proximal cueing (N = 10, W = 28; p = 0.016) (Figure 2; see Table 1 for individual performance data). In contrast, the monkeys performed clearly above chance level in all three proximal *test* conditions (N = 10, doll & human both: W = 55, p = 0.002; stick: W = 45, p = 0.004) and better than chance in the distal stick condition (N = 9, W = 24, p = 0.047), but in none of the other distal conditions (N = 9, doll: W = 13, p = 0.219; human: W = 11, p = 0.461; see Table 1 for individual performance data).

**Table 1:** Percentage of correct choices in each condition for each monkey.

Subject	Baseline	Doll	Human	Stick	Re-Test
**PROXIMAL**					
Ismael	**77.78**	**100.00**	**94.44**	66.67	**88.89**
Lenny*	**77.78**	**83.33**	**72.22**	66.67	**83.33**
Linda	55.56	**94.44**	**88.89**	**94.44**	**77.78**
Maja*	61.11	**88.89**	**72.22**	**83.33**	66.67
Paule	50.00	61.11	55.56	50.00	50.00
Popey*	50.00	66.67	66.67	**77.78**	55.56
Selina	61.11	**83.33**	66.67	**72.22**	**83.33**
Sophie*	61.11	**83.33**	**88.89**	**77.78**	**77.78**
Sunny	50.00	61.11	61.11	55.56	50.00
Susi* 1	66.67	66.67	55.56	61.11	
***Mean***	***61.11***	***78.89***	***72.22***	***70.56***	***70.37***
**DISTAL**					
Ismael*	**83.33**	66.67	44.44	**72.22**	55.56
Lenny	44.44	44.44	66.67	44.44	**72.22**
Linda*	50.00	55.56	55.56	55.56	66.67
Maja	61.11	**72.22**	66.67	**72.22**	44.44
Paule*	66.67	50.00	50.00	50.00	50.00
Popey	50.00	50.00	44.44	77.78	50.00
Selina*	50.00	**72.22**	38.89	66.67	55.56
Sophie	55.56	44.44	72.22	50.00	66.67
Sunny*	50.00	50.00	44.44	55.56	55.56
***Mean***	*56.79*	*56.17*	*53.70*	***60.49***	*57.41*

The mean values for the proximal and distal conditions are written in italic, significant performances in bold.

1 due to health problems, this monkey participated in the proximal trials only.

*subjects started with the respective conditions (i.e. proximal or distal).

In the baseline and test conditions, Friedman ANOVAs revealed a significant difference between the conditions with proximal (N = 9, χ^2^ = 14.27, df = 3, p = 0.003) but not with distal cueing (N = 9, χ^2^ = 2.28, df = 3, p = 0.516). Post-hoc tests in the proximal conditions revealed a significant difference between the baseline and doll condition (p<0.001) and the baseline and human condition (p = 0.033), but no significant difference between the baseline and the stick condition (p = 0.095). There were no significant differences between the different cue-providing elements within these test conditions (human vs. doll: p = 0.366; stick vs. human: p = 0.608; stick vs. doll: p = 0.215).

**Figure 3:**
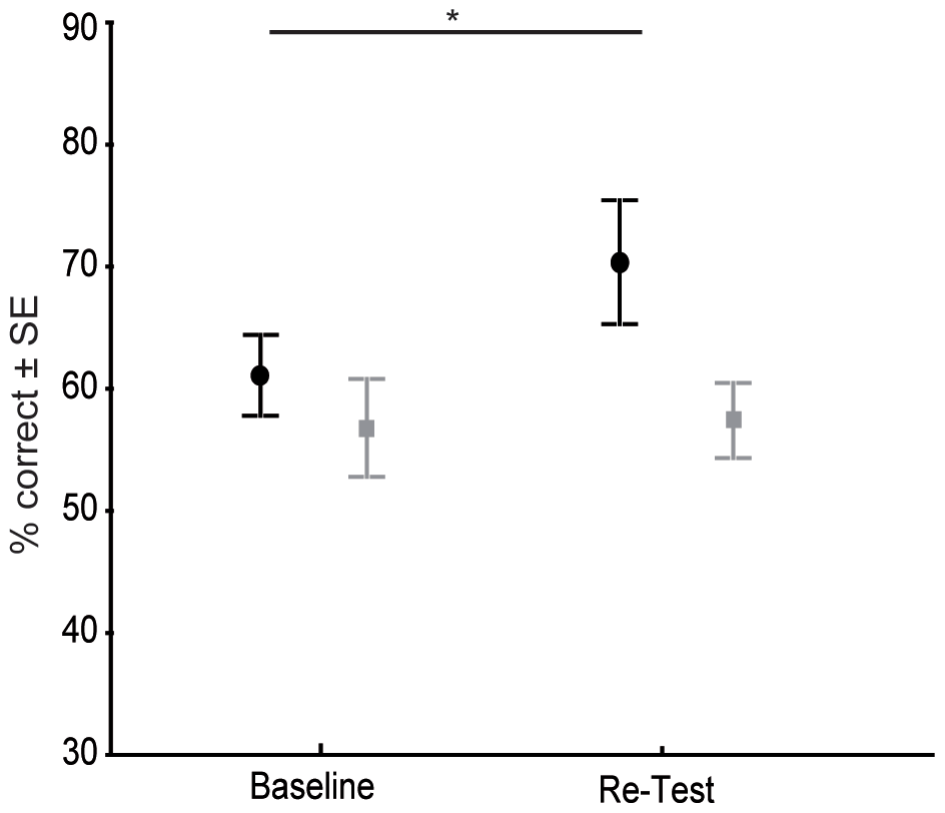
Comparison of the Baseline and Re-Test conditions. The figure shows the mean percentage of correct trials in the Baseline and Re-test conditions (black circle: proximal; grey square: distal). The performance in the proximal Re-Test condition was significantly better than in the proximal Baseline condition (Wilcoxon's exact test, *p = 0.016).

When we repeated the baseline condition in the re-test at the end of the experiment, the monkeys' performance in the proximal condition improved to 70.4% correct choices, which is a significant increase when compared to the proximal baseline condition (N = 9, W = 28, p = 0.016) (Figure 3). Although the performance of the monkeys was significantly above chance in the distal re-test with 57.4% correct choices (N = 9, W = 23, p = 0.047), this was no significant improvement compared to the distal baseline condition (N = 9, W = 2, p = 0.945).

## Discussion

Our results show that the performance of the monkeys increased significantly when the experimenter stood behind the curtain and only the pointing cue was visible to the subjects, suggesting that the presence of the experimenter indeed distracted the monkeys in the normal object-choice pointing test. This may be due to two aspects: first, the visual absence of the experimenter may have reduced potential effects of fear (as they may have perceived the experimenter as a competitor), and second, the pointing cue may have been more conspicuous when the rest of the person stood behind the curtain.

We believe that our findings are in line with the distraction hypothesis proposed by Mulcahy and Call [6], but suggest that additional distracting factors can influence cue saliency. It seems that not only the close proximity of the two containers can distract nonhuman primates from using human communicative cues, but also the presence of the experimenter herself. When only the cups and relevant cue features were visible during the test, the performance of the long-tailed macaques increased significantly, despite a relatively close distance between the cups. In fact, not all apes succeeded in studies using the peripheral paradigm proposed by Mulcahy and colleagues (e.g. [36]), indicating that the distance between the cups cannot be the only explanation for their failure.

Our results further suggest that social inhibition may in part explain the failure of primates to use human cues. When the experimenter was hidden behind the curtain, the performance improved considerably, supporting the hypothesis that the monkeys might have been afraid of the experimenter's appearance. Unfortunately, we could not systematically analyse fear responses in our monkeys as these rather subtle reactions were not visible from the videos, but future experiments could try to have a closer look at this potential confounding factor. Even though the experimenter's entire body was hidden in our experiments, the results of previous studies suggest that monkeys are most responsive to the orientation of the head and the eyes; for instance, nonhuman primates preferentially steal food from an experimenter not facing the animals [29], [37], [38]. To examine whether the head and eyes are the most distracting body parts, future studies could include a condition in which the human is pointing at the baited cup but is oriented away from the monkey.

This finding also contributes to the discussion on the superior performance of dogs and other domesticated or trained species in using human communicative cues. Some researchers suggested that through domestication dogs did not acquire special socio-cognitive skills but that learning was facilitated, especially through the reduction of fear and an increased tolerance to the sight of humans [33], [34], [39], [40]. In consequence, dogs may just be better adapted to the specific context in a pointing situation than nonhuman primates. Likewise, highly trained animals and enculturated apes who have passed the pointing task may have fewer problems facing a human, thereby increasing their attention to the actual task [13], [14], [18].

Furthermore, we are positive that the procedure, and not learning through a relatively high number of trials during the course of the experiment accounted for the increased performance. We did not see an increase in performance within the first baseline sessions (e.g. 62.9% correct in the first proximal baseline session, but only 58% correct in the second session), but the monkeys performed immediately better in the following test conditions (over 75% correct in the first proximal test session). Similarly, also in the studies of Mulcahy and colleagues [6], a sudden increase in performance occurred when switching to the more distal condition, arguing against simple learning to use the cue due to repeated trials. Previous studies testing monkeys with similar distances between the cups as we used, i.e. placing the cups on one board in front of the monkeys, also indicate that experience with the pointing gesture alone is not enough to learn to respond to the cue, at least within the amount of trials administered in our study. For instance, Anderson and colleagues [41] tested capuchin monkeys and found no increase in performance over 300 training trials; only one monkey learned to use the point after adding a gaze cue, whereas the other monkeys still performed at chance level after more than 600 trials. It is unclear if in our study the use of different objects to point at the baited cup without hiding the experimenter, would have been sufficient to increase the subjects' attention to the cue. Unfortunately, we did not have a sufficient number of naïve subjects to test this hypothesis. Future studies could use the same procedure, i.e. pointing at a cup with sticks, artificial arms, etc., without hiding the experimenter, which would allow disentangling which manipulations help the animals to use a pointing cue.

In some previous object-choice studies, the experimenter pointed *and* gazed at the correct location to enhance the communicative context of the experiment. More specifically, it was argued that establishing eye contact is a necessary prerequisite to effectively use a pointing cue (see [12]). The combination of different cues, however, makes it impossible to disentangle their respective influences [6], [42], [43]. Importantly, under some circumstances chimpanzees may rely on gaze, but not on point cues in object-choice tasks (see also [44]). In our study, the long-tailed macaques did use the pointing cue without additional gaze cues, questioning the assumption that this is an essential prerequisite to perform successfully. In fact, the monkeys were able to use the pointing cue without any additional communicative act. The kind of cue used to point at the correct location, i.e. whether it was “relatively social” (human), “abstract social” (doll) or “abstract arbitrary” (stick) did not have a major influence on the monkeys' performance. Instead, local enhancement may account for much of the monkeys' performance, as they performed significantly better when the distance between the cue and the cup was close. Similarly, recent dog studies comparing physical and social cues also found no specific tuning towards social cues and even report that dogs that had learned to use a physical cue to find food had problems to use social cues afterwards. This stimulus interference is a typical characteristic of associative learning [31], [32]. This suggests that for the use of a human pointing gesture, a special understanding of its communicative intent is not always necessary. Instead associative learning mechanisms may be sufficient.

Similarly, if the monkeys had understood the communicative intent, they should have performed equally in the distal and the proximal conditions, as the distance between the cup and gesture should not matter that much, but this was not the case (see [26] for similar findings in chimpanzees). Although one monkey used a distal cue in the re-test condition, the mean performance of the monkeys reached only approx. 57% correct choices and it remains a question of empirical investigation whether they would learn to reliably use the distal pointing cue when subjected to extensive training. Interestingly, three monkeys performed above chance in the distal stick condition. Possibly the stick provided the most distinct cue as it was thinner and straighter as the arms, allowing the monkeys to better follow the direction it pointed at. It seems that these basic cue features only become relevant when the distance between the cue and the cup is large as there were no significant differences between the three cue-providing elements in the proximal conditions.

Without additional gazing, proximal pointing is the cue most successfully used by nonhuman primates (e.g. [41], in [26] only one chimpanzee used a distal pointing cue). Furthermore, also dogs prefer to choose the container closer to the human pointing gesture [45] suggesting that the subjects may initially learn that a close spatial association between the cue and the cup indicates the place of the reward. In children, it is also not fully clear to what extent learning accounts for the initial utilisation of pointing. Twelve-month-old infants look in the direction of the pointing, but only 15-months olds show some understanding of the underlying communicative intent ([46], [47], see also [48] for a comparison of dogs' and human infants' understanding of pointing).

Taken together, when distractors are removed, long-tailed macaques learn relatively quickly to use human pointing cues to locate hidden food, and they can transfer this knowledge to more complex situations, i.e. when the human is visible again. Reducing the ambiguity not only of the cues but of the entire experimental situation apparently facilitates the usage of human communicative cues in nonhuman primates.

In sum, our results suggest that the presence of the experimenter can distract nonhuman primates in the object-choice task. Furthermore, in our study simple local enhancement was sufficient to associate the cue with the baited cup. There was no difference in performance concerning social vs. non-social cues, suggesting that the social aspect may not play a major role in the acquisition of this association. These findings highlight the importance of the test conditions, and question the assumption that species-specific differences in the object-choice task are only due to cognitive dissimilarities.
